# One-staged combined hip and knee arthroplasty: retrospective comparative study at mid-term follow-up

**DOI:** 10.1186/s13018-019-1337-0

**Published:** 2019-09-05

**Authors:** Stefano Petrillo, Matteo Marullo, Michele Corbella, Paolo Perazzo, Sergio Romagnoli

**Affiliations:** 1 Prosthetic Surgery Centre, IRCCS Orthopedic Institute Galeazzi, via Riccardo Galeazzi 4, 20161 Milan, Italy; 2 Anaesthesiology and Intensive Care Unit, IRCCS Orthopedic Institute Galeazzi, via Riccardo Galeazzi 4, 20161 Milan, Italy

**Keywords:** Hip arthroplasty, Knee arthroplasty, One-staged, Outcomes

## Abstract

**Background:**

To date, few knowledge is available about safety and effectiveness of one-staged combined hip and knee arthroplasty. The aim of our study was to evaluate, in a comparative fashion, complications and outcomes in patients who underwent one-staged hip and knee arthroplasty.

**Methods:**

Forty-two patients were enrolled and allocated into two groups of 21 patients each: one-staged hip and knee arthroplasty (group A) and two-staged hip and knee arthroplasty (group B). The follow-up averaged 50.2 months. Postoperative complications and implant survivorship were assessed prospectively. Outcomes were evaluated with Harris Hip score (HSS), Western Ontario Mc-Ministry score for the hip (h-WOMAC), Knee Society score (KSS), and Western Ontario Mc-Ministry knee score (k-WOMAC). Hip and knee range of motion (ROM) were measured both preoperatively and at the last follow-up.

**Results:**

Two (9.5%) patients in group A and three (14.3%) patients in group B developed complications (*P* = 0.8). Although a significant decrease in postoperative haemoglobin (Hgb) values was found in group A patients during the hospital stay, no differences in blood transfusions were found (*P* = 0.8). No significant differences were found comparing clinical-functional outcomes between the two groups, while a significant reduction of hospital length of stay was shown in group A patients.

**Conclusions:**

One-staged combined hip and knee arthroplasty could be considered in patients with co-existing severe hip and knee osteoarthritis, providing similar complications and mid-term outcomes of two-staged procedures. However, the reproducibility safety and reliability of these procedures should be confirmed in prospective comparative randomised trials with more numerous patients.

**Trial registration:**

Retrospectively registered

## Background

Osteoarthritis (OA) of the hip and knee is the fourth and eighth most frequent pathology, respectively, in males and females, producing elevated costs for the National Health systems [1]. In population over 60 years, the prevalence of hip OA (h-OA) is 4.2% while the prevalence of knee OA (k-OA) is 10.1% [2]. However, a significantly increased incidence of both h-OA and k-OA was shown in obesity, metabolic syndrome, rheumatoid arthritis, hematologic disorders, immunological disease, and in former professional athletes [3–6].

Since 1970s, several authors have reported the results of simultaneous bilateral hip or knee arthroplasty procedures. First studies demonstrated high complication rates of one-staged bilateral hip or total knee arthroplasty (TKA) than staged procedures [7, 8]. However, Salvati et al. [9] and Ritter et al. [10] reported, respectively, satisfactory outcomes of bilateral hip or knee replacement surgery performed in one-stage setting. More recently, Romagnoli et al. [11, 12] have shown no differences in complications, revisions, and transfusion rates in patients undergoing one-staged bilateral or unilateral hip or knee arthroplasty.

Despite there is huge information about bilateral one-staged hip or knee replacement procedures, and to date more than 160 articles were published on these topics, there is few knowledge about outcomes, complications, and implant survivorship of one-staged combined hip and knee arthroplasty. Only one study [13] was found in the literature reporting high complication rate and implant survivorship of 94% at 3 years of follow-up. Moreover, the results shown in the same study refer to prosthetic materials and designs as well as anaesthetics procedures of over 15 years ago, which are certainly less reliable with respect to the most modern.

The objective of our study was to evaluate complications, clinical and functional outcomes, and implant survivorship in a consecutive series of patients who underwent one-staged hip and knee arthroplasty and to compare such results with those obtained in a matched-pair control group of patients who underwent two-staged hip and knee arthroplasty.

## Methods

The study was approved by the ethics committee board of our Institution. According to the Helsinki Declaration, each patient enrolled in the study gave his informed consent to participate.

### Patients enrolment

The database of surgical procedures performed by the senior author (S.R.) was analysed the 3 September 2017 to identify patients who underwent both hip and knee arthroplasty. Patients operated from 1 January 2014 to 3 September 2017 were excluded, obtaining only patients with a minimum follow-up of 36 months. During the index period, 25 patients underwent one-staged hip and knee arthroplasty, while only 4 (15.4%) of them refused our invitation, remaining 21 patients available for the present investigation. A match-paired group by gender, age, body mass index (BMI), and duration of follow-up of 21 patients who underwent two-staged hip and knee arthroplasty within 1 year from the first operation was selected from the same surgical records database. At the end, 42 patients were available at an average follow-up of 50.2 months. The patients were divided into two groups of 21 patients each, according to the type of surgery received: one-staged hip and knee arthroplasty (group A) and two-staged hip and knee arthroplasty (group B).

The indications for one-staged hip and knee arthroplasty were as follows: radiographic evidence of severe OA of both hip and knee: Kellgren Lawrence (KL) grade 3 or higher; impairment of walking capacitates (walking autonomy lower than 100 m), referred reduction of the quality of life due to hip and knee pain and loss of function (impairment of activities of daily living); and motivation and compliance of the patients sustaining a combined procedure.

The following data were extracted from the medical records of the patients: (1) age, (2) sex, (3) weight, (4) height, (5) BMI, (6) affected limb (right or left or both), (7) preoperative diagnosis, (8) date of the surgical procedure, (9) American Society of Anesthesiology (ASA) score, (10) type of hip prosthesis, (11) type of knee prosthesis, (12) variation of haemoglobin (Hgb) values during hospitalisation, (13) perioperative complications, and (14) discharging information.

Total hip arthroplasty (THA) procedures were performed in all patients through a minimally invasive posterior approach. All the enrolled patients except one received the same uncemented THA implant: trabecular metal cup (Trilogy acetabular system; Zimmer-Biomet; Warsaw-USA), MIS stem (Fitmore hip stem; Zimmer-Biomet; Warsaw-USA), 32 mm ceramic head (Biolox delta; Ceramtech; Plochingen-Germany), and a polyethylene liner. The remaining patient of group A underwent revision of a THA stem with a cementless stem (CLS-Spotorno; Zimmer-Biomet; Warsaw-USA).

**Fig. 1:**
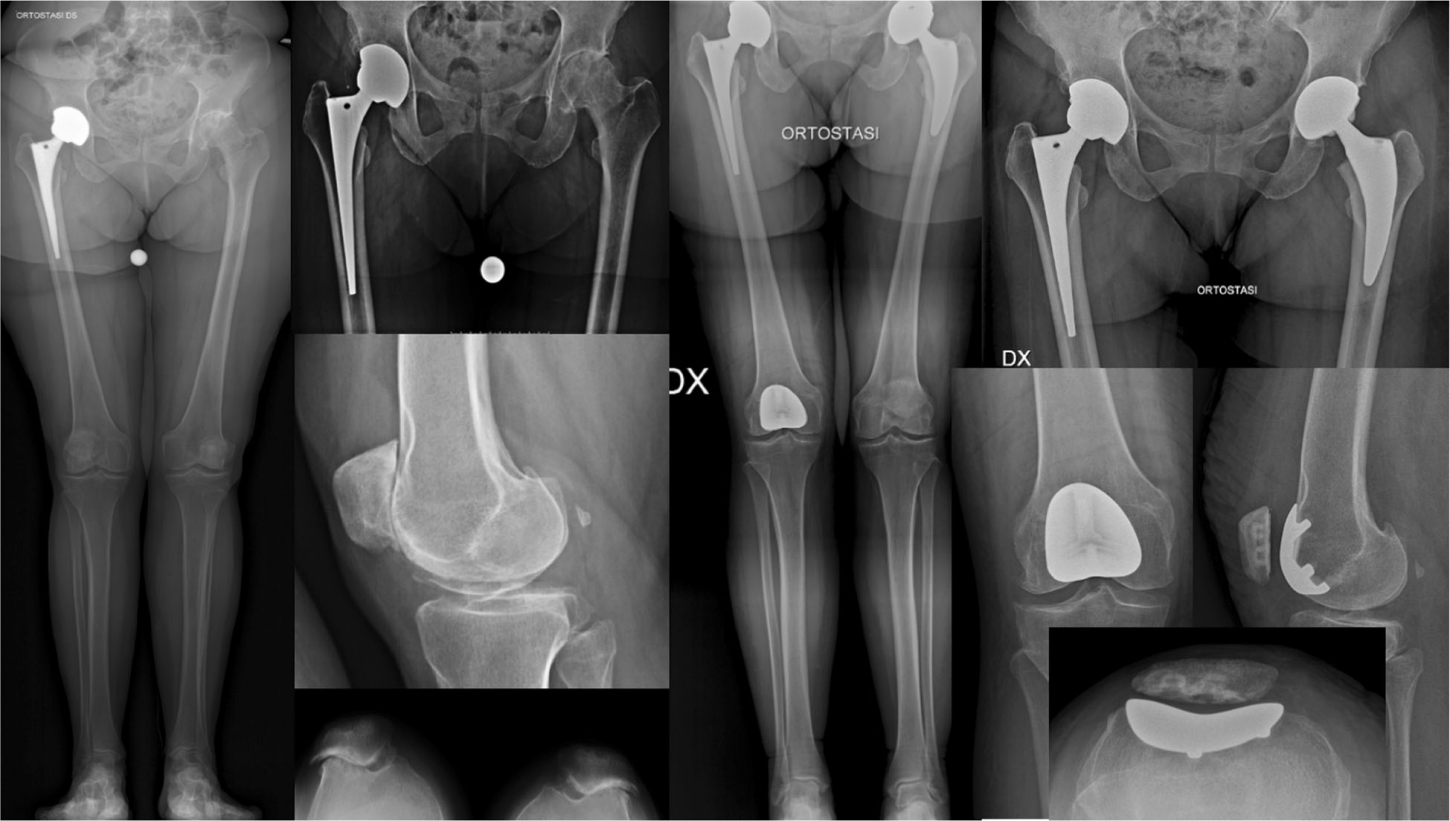
One-staged combined THA and PFJ arthroplasty

**Fig. 2:**
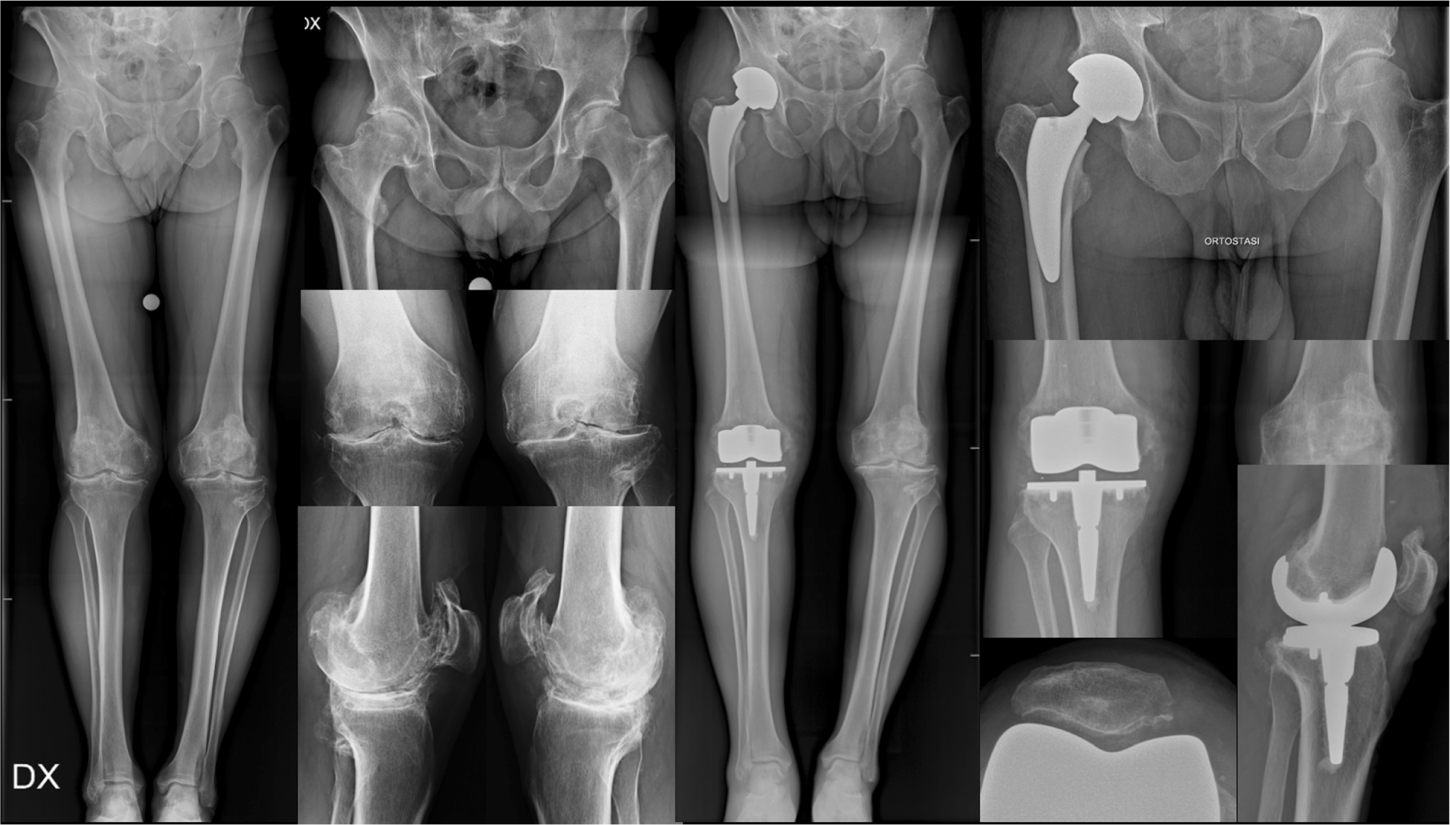
One-staged combined THA and TKA

In all patients, UKAs, patellofemoral joint (PFJ) arthroplasties (Fig. 1), and TKAs (Fig. 2) were cemented and performed through a mini mid-vastus approach. A fixed bearing UKA (Zimmur Unicompartmental Knee; Zimmer-Biomet; Warsaw-USA) was implanted in 12 (57.1%) patients of each group, while a PFJ arthroplasty (PFJ Gender, Zimmer-Biomet; Warsaw-USA) was used in 2 (9.5%) and 1 (4.8%) patients of group A and B respectively. Only one (4.8%) patient of group A received a bi-compartmental knee arthroplasty, using a Zuk UKA for medial the compartment and an Allegretto UKA for lateral compartment (Allegretto unicompartmental knee arthroplasty, Zimmer-Biomet; Warsaw-USA). A mobile bearing TKA (Innex total knee, Zimmer-Biomet; Warsaw-USA) was implanted in 6 (28.6%) and 8 (38.1%) patients respectively (Table 1).

**Table 1:** Demographic details of the patients

Demographic details of the patients			
	Group A	Group B	*P*
Sex (male:female)	11:10	09:12	0.5
Age at surgery (years)	69.4 ± 7.9	70.1 ± 10.2	0.6
BMI (kg/m^2^)	26.9 ± 3,9	28,2 ± 4.6	0.07
Mean follow-up (months)	42.8 ± 15.6	53.6 ± 12.5	0.06
Preoperative Hgb (g/dl)	14.2 ± 1.2	13.7 ± 1.2	0.6
Preoperative diagnosis			
Primary OA	18 (85.7%)	19 (90.5%)	0.9
Rheumatoid arthritis	2 (9.5%)	2 (9.5%)	0.9
Revision hip arthroplasty	1 (4.8%)	0	n.a.
Revision knee arthroplasty	0	0	n.a.
Surgical procedure			
Ipsilateral	14 (66.6%)	10 (47.6%)	0.9
Contralateral	7 (33.4%)	11 (52.4%)	0.8
ASA score			
ASA 2	20 (95.2%)	19 (90.5%)	0.9
ASA 3	1 (4.8%)	2 (9.5%)	n.a.
Type of knee arthroplasty			
Medial UKA	12 (57.1%)	12 (57.1%)	0.9
Bi-UKA	1 (4.8%)	0	n.a.
PFJ arthroplasty	2 (9.5%)	1 (4.8%)	n.a.
TKA	6 (28.6%)	8 (38.1%)	0.9

Results reported as mean value + standard deviation

*P* results of Mann-Whitney *U* test or *χ*^2^ test, *BMI* body mass index, *Hgb* haemoglobin, *OA* osteoarthritis, *ASA* American Society of Anesthesiology, *UKA* unicompartmental knee arthroplasty, *PFJ* patellofemoral joint, *TKA* total knee arthroplasty, *n.a.* not applicable

All surgical procedures, both in study and control group, were performed under combined spinal-epidural anaesthesia using 12.5 mg of levobupivacaine 0.5%. Tranexamic acid (TXA) (1 mg/kg) was used 10 min preoperatively, and the same dosage was administered 5 h after surgery at 50 ml/h in 250 ml of 0.9% sodium chloride solution. Moreover, 4 mg of dexamethasone was administered both preoperatively and every 24 h for the first 2 days after surgery. In group A patients, an epidural catheter was positioned prior to surgery, and 48 h after surgery was used to administer 5–7 ml of ropivacaine 0.2%. After 2 days from the index surgery, in all patients, 10 mg/5 mg of oxycodone/naloxone was administered at 12 h intervals for other 2 days.

In all patients, supervised rehabilitation was performed at the Department of Physical Medicine of our Institution. The patients were discharged only when they had reached such a degree of autonomy that they could manage to ascend and descend the stairs, walk for more than 100 m, take care of their personal hygiene, and present Hgb values higher than 10 g/dl.

### Clinical evaluation

Clinical outcomes were assessed at the final follow-up by an independent examiner (S.P.) who was not involved in surgical procedure and included Harris Hip score (HSS) [14], Western Ontario Mc-Ministry score for the hip (h-WOMAC) [15], Knee Society score (KSS) [16], and Western Ontario Mc-Ministry knee score (k-WOMAC) [17, 18].

### Functional evaluation

Functional outcomes were assessed in a prospective fashion (preoperative and at last follow-up) by an independent examiner (S.P.) who was not involved in the surgical procedure. Regarding the hip, the following range of motion (ROM) was measured with a medical goniometer: flexion, internal rotation, external rotation, abduction, and adduction.

Regarding the knee, the following ROM was measured with a medical goniometer: extension, flexion, and extension deficit. The presence of any extension deficits was also considered.

### Radiographic evaluation

Radiographic evaluation was performed at the final follow up and included standard X-ray of the hip (Ap view and frog-leg view) and knee (Ap view, lateral view, Merchant view).

### Statistical analysis

Statistical analysis was performed using SPSS for Mac (IBM SPSS Statistics Desktop version 22.0; Chicago-Illinois). For quantitative and normally distributed measurements, the mean value and standard deviation were calculated. The independent variables considered were the following: age, sex, BMI, and follow-up. The results considered were as follows: HHS, h-WOMAC, KSS, k-WOMAC, and preoperative and postoperative ROM of the hip and knee. The comparison between the two groups for each independent variable was performed with the Mann-Whitney *U* test for continuous variables, while the *χ*^*2*^ test was used for the categorical variables. The results considered (HHS, h-WOMAC, KSS, KSS-f, k-WOMAC, ROM of the hip and knee) were compared with the Mann-Whitney *U* test. *P* values lower than 0.05 were considered statistically significant. Statistical analysis of implants survival was performed using the Kaplan-Meier method, while a post hoc power analysis was performed with G-Power software (GPower 3.1).

## Results

### Demographics

The demographic characteristics of the patients enrolled in the study are reported in Table 1. The follow-up averaged 42.8 months in group A and 53.6 months in group B. No statistically significant differences were found comparing age, sex, BMI, follow-up duration, and preoperative Hgb value between the two groups of patients.

### Complications

No infections or thromboembolic events occurred. No implant failure or revisions were detected. Two (9.5%) patients in group A and three (14.3%) patients in group B developed postoperative complications (*P* = 0.8). One (4.8%) patient in group A developed a surgical wound infection, treated with antibiotics and resolved within 1 month of surgery, while the other patient (4.8%) developed a urinary tract infection treated with antibiotics. Two post-traumatic hip dislocations in two (9.5%) patients in group B occurred at a distance of 6 and 8 months after surgery, respectively, both reduced in the emergency room and managed with hip brace for 1 month, without the need for revision of the implants. The remaining patient (4.8%) in group B had a flexion contraction after a TKA, which resolved with an extra-period of rehabilitation.

### Blood loss and transfusions

The variation of Hgb is reported in Table 2. A statistically significant decrease of Hgb values was found in group A patients 24 h after surgery and during all hospital stay. Five transfusions were performed in three (14.3%) patients in group A, and three transfusions in two (9.5%) patients in group B (*P* = 0.6).

**Table 2:** Surgery-related information

Surgery-related information			
	Group A	Group B	*P*
Mean duration of combined surgery (min)	62 ± 14.2	66 ± 15.3	0.6
Mean Hgb 24 hrs after surgery (g/dl)	11.1 ± 1.1	11.9 ± 1.4	0.4
Hgb at discharge (g/dl)	10 ± 0.8	10.6 ± 1.5	0.4
Mean Hgb loss 24 hrs after surgery (g/dl)	3.1 ± 0.9	1.8 ± 0.7	0.0002*
Mean Hgb loss during hospitalisation (g/dl)	4.3 ± 1.4	3.1 ± 1.3	0.01*
Length of hospital stay (days)	15.5 ± 2.6	27.6 ± 10.1	0.006*

Results reported as mean value ± standard deviation

*Hgb* haemoglobin, *hrs* hours, *P* result of Mann-Whitney *U* test

*Statistically significant

### Surgery duration and hospitalisation

A statistically significant reduction of hospital stay length was found in group A (Table 2). The average duration of combined surgery was 62 ± 14.2 min (range 45–90 min) and 66 ± 15.3 min (range 50–95 min) in group A and B patients respectively.

### Clinical and functional outcomes

At the last follow up, no statistically significant differences were found comparing clinical and functional outcomes between the two groups (Table 3). Moreover, a statistically significant improvement in all functional outcomes was found at the last follow up (Table 4).

**Table 3:** Clinical and Functional outcomes

Clinical and functional outcomes			
	Group A	Group B	*P*
Hip			
HSS	96.6 ± 4.1	95.8 ± 5.2	0.8
h-WOMAC	94.1 ± 5.8	97.2 ± 3.7	0.07
Flexion	111.9° ± 8.7°	115.3° ± 9.9°	0.2
Internal rotation	36.6° ± 6.7°	35° ± 8.6°	0.6
External rotation	41.2° ± 4.4°	43.6° ± 2.9°	0.09
Adduction	40.7° ± 4.3°	37° ± 7.5°	0.2
Abduction	38.1° ± 5.8°	40.3° ± 6.9°	0.1
Knee			
KSS	93.1 ± 4.5	94 ± 6.8	0.1
KSS-function	92.4 ± 7	91 ± 9.7	0.8
k-WOMAC	91.9 ± 4.2	91.5 ± 11.3	0.1
Extension	117.3° ± 9.4°	119.3° ± 12.6°	0.3
Flexion	0.3° ± 1.2°	0.2° ± 1.3°	0.8

Results reported as mean value + standard deviation

*HSS* Harris Hip score, *h-WOMAC* Western Ontario Mc-Ministry score for the hip, *KSS* knee society score, *k-WOMAC* Western Ontario Mc-Ministry score for the knee, *P* result of Mann-Whitney *U* test

**Table 4:** Functional outcomes

Functional outcomes						
	Group A			Group B		
	Preoperative	Postoperative	*P*	Preoperative	Postoperative	*P*
Hip						
Flexion	61.4° ± 35.8°	111.9° ± 8.7°	< 0.00001	33.5° ± 18.4°	115.3° ± 9.9°	< 0.00001
Internal rotation	2.1° ± 4.1°	36.6° ± 6.7°	< 0.00001	1.7° ± 4.5°	35° ± 8.6°	< 0.00001
External rotation	13.6° ± 8.1°	41.2° ± 4.4°	< 0.00001	11.5° ± 5.1°	43.6° ± 2.9°	< 0.00001
Adduction	11.6° ± 5.5°	40.7° ± 4.3°	< 0.00001	11° ± 7.2°	37° ± 7.5°	< 0.00001
Abduction	12.4° ± 9.8°	38.1° ± 5.8°	< 0.00001	14.3° ± 8.6°	40.3° ± 6.9°	< 0.00001
Knee						
Flexion	115.2° ± 6°	117.3° ± 9.4°	< 0.00001	99.7° ± 29.7°	119.3° ± 12.6°	< 0.00001
Loss of extension	1.6° ± 2.8°	0.3° ± 1.2°	< 0.00001	3.9 ± 4.9°	0.2° ± 1.3°	< 0.00001

Results reported as mean value ± standard deviation

*P* result of Mann-Whitney *U* test

## Discussion

The aim of our study was to compare complications, outcomes, and implant survivorship in patients who underwent one-staged or two-staged combined hip and knee arthroplasty. To our knowledge, no comparative studies have been published yet on the topic.

One-staged hip and knee arthroplasty procedures have the same complications rate of staged procedures, with similar clinical and functional outcomes at a mid-term follow-up. Furthermore, a fully superimposable implant survivorship was found. However, in patients receiving the one-staged procedure, a significant reduction of Hgb values was measured 24 h after surgery and during all the hospitalisation period. This reduction was approximately 1 g/dl higher than that found in the control group, and the average postoperative loss of Hgb was 4.3 g/dl in the study group and 3.1 g/dl in controls. Nevertheless, the transfusion rate was 14.2% in the study group and 9.5% in controls, and this difference was not statistically significant. From the analysis of patient discharge information, a statistically significant difference emerged between the two groups regarding the length of hospital stay. Indeed, hospitalisation length calculating both surgical and rehabilitation department period averaged 15.5 days in the study group and 27.2 days in controls.

It was difficult to compare our results with those reported in the literature because we found only one study on the topic, which was published in 2002 [13]. The authors of such study reported a complication rate ranging from 16.7 to 19.5%, three deaths within 1 year of surgery, and an implant survival of 94% and 83% at 3 and 5 years respectively [13]. In our series, 9.5% of complications were found in the study group, but only 4.8% of these were related to surgery (one case of surgical wound infection of the hip). Nevertheless, no deaths occurred at an average follow-up of 42.8 months, with an implant survivorship of 100%.

We believe that this discrepancy between our results and those reported by Ritter et al. [13] could be related to preoperative indication, patients’ selection, surgical technique, and anaesthetic management. Ritter et al. [13] included patients with a mean age of 71 years, and 9% and 11% of them underwent hip and knee arthroplasty revision respectively. In contrast, in our study, the mean age of the study group patients was 69 years, and we included only 4.8% of hip arthroplasty revisions and no case of knee arthroplasty revisions.

Considering surgical technique, all THAs were performed with a minimally invasive posterior approach, sparing extra-rotators muscle-tendon group, and incising only the piriformis tendon. In this manner, the risk of bleeding due to incision of the quadratus of the femur, which is in close contact with the circumflex arteries anastomosis, can be reduced. Moreover, the use of a short stem maintains greater bone stock and significantly reduces bleeding compared to the use of more invasive straight stems [19–22].

The 71.4% of our knee arthroplasty were small implants (UKA or PFJ arthroplasty). It is well known that the choice of “small implants” reduces operating time as well as intraoperative and postoperative bleeding [23–26]. Moreover, all knee arthroplasties were performed without tourniquet and with a “free-hand” technique, without using instrumentations and cutting guides, reducing, in this manner, operating time and intraoperative-postoperative bleeding [27, 28], as well as invasiveness on the bone tissue avoiding femoral and tibial intramedullary canal violation.

It is well known that surgery duration and postoperative bleeding are two crucial aspects influencing the length of hospital stay [29, 30]. In patients who underwent one-staged hip and knee arthroplasty, the mean operating time was 62 min, which can be considered an acceptable duration of surgery, especially for this kind of procedures. Moreover, the use of TXA, both preoperatively and during the first 5 h after surgery, reduced significantly the perioperative bleeding. However, we are aware that TXA does not decrease the need for blood transfusion after both hip and knee arthroplasty.

The main strength of our study was that all surgical procedures were performed by the same surgeon with over 30 years of experience in hip and knee prosthetic surgery, using a well-standardised surgical technique for both interventions. Furthermore, all evaluations were performed blindly by an orthopaedic surgeon who was not involved in the surgery. Another strength is that no differences were present concerning the anthropometric characteristics of the patients, demonstrating that the sample chosen was appropriate for the present investigation.

Limitations should be underlined when considering our results. The most important one is the retrospective design of the study, with a limited sample size. However, we have analysed the outcomes of a quite rare surgical procedure, representing less than 0.5% of our 1200 annual prosthetic surgeries. At the same time, we have described an interesting sample of patients with a co-existing hip and knee pathology which, although not rare itself, is unusual when both are deemed severe enough to warrant arthroplasty at the same setting. Moreover, a post hoc power analysis was performed, showing that with the sample of patients enrolled, our study has an acceptable strength (1-beta = 0.4). Finally, with an average follow-up of 50.2 months, our outcomes and implant survivorship data cannot be considered as univocal. Another limitation is the huge heterogeneity of knee implants used (UKA, PFJ arthroplasty, and TKA).

In conclusion, in view of the limitations above, we believe that one-staged combined THA and knee prosthesis is safe and effective in patients with combined h-OA and k-OA, and it is convenient for the patient, because of the single surgical and anaesthetic procedure, as well as the rehabilitation period. Moreover, the shorter duration of hospital stay in the study group patients represents an advantage for both patients and Institutions in terms of reduction of costs and hospitalisation-related complications.

However, we are aware that the results reported in the present investigation were found in a small sample of patients. Moreover, they refer to the activity of a single surgeon with great expertise in hip and knee prosthetic surgery, working in a high-volume hospital specialised in this type of surgery, and therefore, the reproducibility, safety, and reliability of these procedures should be confirmed with further randomised trials with more numerous samples of patients and with longer follow-up.

## Conclusion

One-staged combined hip and knee arthroplasty could be considered in patients with co-existing severe hip and knee osteoarthritis. In the present investigation, similar outcomes, complications rate, and implant survivorship was found at a mid-term follow-up in patients undergoing one-staged or staged hip and knee arthroplasty surgery. However, in order to reach definitive conclusions, the safety and reliability of these procedures should be confirmed in prospective comparative randomised trials with more numerous patients.

## Data Availability

None.

## Abbreviations

ASA: American Society of Anesthesiology
BMI: Body mass index
Hgb: Haemoglobin
h-OA: Hip OA
HSS: Harris Hip score
h-WOMAC: Western Ontario Mc-Ministry score for the hip
KL: Kellgren Lawrence
k-OA: Knee OA
KSS: Knee Society score
k-WOMAC: Western Ontario Mc-Ministry knee score
OA: Osteoarthritis
PFJ: Patellofemoral joint
ROM: Range of motion
THA: Total hip arthroplasty
TKA: Total knee arthroplasty
UKA: Unicompartmental knee arthroplasty
